# A novel invasive *Streptococcus pyogenes* variant sublineage derived through recombinational replacement of the *emm12* genomic region

**DOI:** 10.1038/s41598-023-48035-2

**Published:** 2023-12-06

**Authors:** Yvette Unoarumhi, Morgan L. Davis, Lori A. Rowe, Saundra Mathis, Zhongya Li, Sopio Chochua, Yuan Li, Lesley McGee, Benjamin J. Metcalf, Justin S. Lee, Bernard Beall

**Affiliations:** 1 Centers for Disease Control and Prevention, Biotechnology Core Facility Branch, National Center for Emerging and Zoonotic Infectious Diseases, Division Scientific Resources, Atlanta, GA USA; 2 Centers for Disease Control and Prevention, National Center for Immunization and Respiratory Diseases, Division of Bacterial Diseases, Respiratory Diseases Branch, Atlanta, GA USA; 3 Eagle GLobal Scientific, LLC, Atlanta, GA USA

**Keywords:** Microbiology, Molecular biology

## Abstract

Group A streptococcal strains potentially acquire new M protein gene types through genetic recombination (*emm* switching). To detect such variants, we screened 12,596 invasive GAS genomes for strains of differing *emm* types that shared the same multilocus sequence type (ST). Through this screening we detected a variant consisting of 16 serum opacity factor (SOF)-positive, *emm* pattern E, *emm82* isolates that were ST36, previously only associated with SOF-negative, *emm* pattern A, *emm12*. The 16 *emm82/*ST36 isolates were closely interrelated (pairwise SNP distance of 0–43), and shared the same *emm82-*containing recombinational fragment. *emm82/*ST36 isolates carried the *sof12* structural gene, however the *sof12* indel characteristic of *emm12* strains was corrected to confer the SOF-positive phenotype. Five independent *emm82/*ST36 invasive case isolates comprised two sets of genetically indistinguishable strains. The *emm82/*ST36 isolates were primarily macrolide resistant (12/16 isolates), displayed at least 4 different core genomic arrangements, and carried 11 different combinations of virulence and resistance determinants. Phylogenetic analysis revealed that *emm82/*ST36 was within a minor (non-clade 1) portion of ST36 that featured almost all ST36 antibiotic resistance. This work documents emergence of a rapidly diversifying variant that is the first confirmed example of an *emm* pattern A strain switched to a pattern E strain.

## Introduction

*Streptococcus pyogenes*, also known as group A streptococci (GAS), is among the most impactful global pathogens. Each year GAS causes millions of non-invasive infections, hundreds of thousands of invasive infections, and hundreds of thousands of cases of post-infectious sequelae such as rheumatic heart disease and glomerulonephritis^1^ . Despite its huge global disease burden, there is still no GAS vaccine. Non-invasive impetigo and pharyngitis infections, as well as asymptomatic skin and throat carriage, provide major reservoirs for systemic infections caused by GAS, which is consistent with highly temporally related mixed clusters shared by both GAS pediatric pharyngitis and invasive infection isolates^2^. Since the start of the U.S. population-based invasive GAS surveillance program Active Bacterial Core surveillance (ABCs) in 1995, type *emm12* has accounted for the third highest number of invasive GAS ABCs cases (7.7% of total), behind *emm89* (7.9%) and *emm1* (18.7%) [unpublished data, see https://www.cdc.gov/abcs/bact-facts-interactive-dashboard.html). During seven year surveillance of pediatric pharyngitis in 10 North American regions, type *emm12* accounted for the second highest number of cases behind *emm1*^3^. A 30 valent multivalent M protein vaccine in development^4^ targeting *emm* types such as *emm12* and crossprotective against non-vaccine M types would be potentially cost-effective, and could decrease key reservoirs for invasive GAS disease and rheumatic heart disease. Theoretically, the effectiveness of such a vaccine could be compromised by recombination between strains resulting in serotype switching events, as has been shown for pneumococci^5^.

GAS are classified into three broad strain types that are distinguishable by the organization (patterns) of *emm* and “*emm-*like” genes that lie downstream of the *mga* transcriptional regulator^6,7^. The majority of pattern A-C strains are pattern A (egs. *emm1, emm3, emm5, emm6, emm12*), defined by lacking *emm* family genes that lie adjacent to *emm*. Pattern A strains account for approximately 40% of invasive GAS cases in ABCs and lack a functional *sof* virulence gene, which also encodes a serum opacifying activity used in classical GAS typing schemes^8,9^. Pattern E strains (egs *emm2, emm4*, *emm11, emm28, emm82*) account for around 55% of invasive GAS ABCs cases and carry active forms of the hypervariable *sof* gene. These variable *sof* sequence types are identifiers that co-vary with *emm* types^9,10^. Pattern D strains are relatively uncommon within the United States, however recently such strains have increased as causes of invasive disease clusters that disproportionally impact disadvantaged adults such as persons experiencing homelessness or who inject drugs^11^. These three broad groups of strains are also distinguished by phylogenetic clustering of full length M proteins, which correlates to conserved binding and structural properties^12^.

Certain unrelated pattern E and D *emm* types are dispersed among unrelated multilocus sequence types (MLSTs), but generally each MLST-defined clonal complex is confined to a single pattern (A-C, D, or E). In contrast to pattern E and D *emm* types, each of the known *emm* types from pattern A-C strains appear to be restricted to a single MLST-defined clonal complex. *emm* types associated with *mga* locus pattern A-C strains (or A-C *emm* cluster, or SOF*-*negative) are more highly associated with the pharyngitis reservoir, pattern D strains are more associated with impetigo, while pattern E strains appear to have equal affinity for both skin and pharyngeal infection sites^6^. This corresponds to seasonal differences within the United States, where pattern E strains have more propensity to cause invasive cases during the warmer months than do pattern A-C strains^13,14^.

Apparently there are boundaries restricting successful strains resulting from genetic transfers of *emm* genes between pattern A-C and pattern E strains. Specifically, SOF*-*negative (pattern A–C) strains have never been observed to carry an *emm* gene corresponding to previously defined E *emm* clusters, and SOF*-*positive strains have not been observed to carry *emm* genes corresponding to A-C clusters. Here we describe a unique and emergent hybrid recombinant strain that carries a pattern E *emm* gene region by virtue of a gene replacement event within a major pattern A-C strain.

## Methods

### Strains

All invasive strains (12,596) recovered since January of 2015- early 2020 through Active Bacterial Core surveillance [ABCs] were subjected to whole genome sequencing during this period with the Illumina platform which serves as the the basis of ABCs strain characterization^11,14^. Strains that were used for phylogenic analysis (684) were all ST36 and included: (i) 648/691 (93.8%) of ABCs *emm12* isolates recovered during 2015–2019; (ii) 16 *emm12* isolates recovered during 2020 from partial year 2020 surveillance; (iii) a previously described^14^ *emm* deletion ST36 strain (20,160,179); (iv) 3 ABCs *emm12* isolates recovered during 2008–2011; (v) All 15 *emm82/*ST36 isolates recovered during 2015–2021 and available as of September 2021 (3 of these—20,214,701, 20,207,673 and 20,154,051—were recovered from non-sterile wound sites); (vi)a single *emm82/*ST36 pharyngitis isolate from which information and short read genomic data was provided by the principal authors of reference^15^.

### Whole genome (short read) sequencing

Genomic DNA extraction and short read whole genome Illumina sequencing was provided for all isolates as previously described^11,14,16^ and deposited in the National Center for Biotechnology Information Sequence Read Archive under BioProjects accession number PRJNA395240. Strains were cultured on Trypticase soy agar supplemented with 5% sheep blood and incubated overnight at 37 °C. Genomic DNA was extracted using a modified QIAamp DNA mini kit protocol (Qiagen, Inc., Valencia, CA, USA). Nucleic acid concentration was quantified by an Invitrogen Qubit assay (Thermo Fisher Scientific Inc., Waltham, MA, USA) and samples were sheared using Covaris M220 or Covaris LE220 ultrasonicators (Covaris, Inc., Woburn, MA, USA) programmed to generate 500-bp fragments. Libraries were constructed on the SciCloneG3 (PerkinElmer Inc., Waltham, MA, USA) using PCR-free library kits (either TruSeq DNA PCR-Free HT library preparation kit with 96 dual indices [Illumina Inc., San Diego, CA, USA], Nugen Ovation Rapid [Tecan Trading AG, Switzerland], or SparQ DNA Library Prep [Quantabio, Beverly, MA, USA]) and quantified by a KAPA qPCR library quantification method (Kapa Biosystems Inc., Wilmington, MA, USA). Whole genome sequence (WGS) was generated with coverage of 50–99 × employing MiSeq instruments and the MiSeq v2 500 or MiSeq v3 600 cycle kits (Illumina Inc). Only ST36 strains (684 total described above) that yielded < 200 genomic sequence contigs (average of 42) were used for phylogenetic analysis. Assembly metrics are summarized in sTable 1. WGS reads for all isolates (12,596) were analyzed by our validated bioinformatics pipeline (https://github.com/BenJamesMetcalf/GAS_Scripts_Reference). The M protein gene type (*emm* type and subtype) defining sequence^17^ was defined by its direct proximity 40 – 50 bp downstream of a highly conserved signal sequence motif defined as “primer 1”^18^. M serotype specific antigens employed in the investigated 30 valent M serotype-specific multivalent vaccine^19^ are encoded by sequences encompassed by or overlapping this *emm* type and subtype defining sequence that consists of 50 codons encoding the N terminus of the M protein. The pipeline output (14,16, sTable 2) included isolate *emm* type, multi-locus sequence type (ST), certain virulence-related determinants, and antimicrobial-resistance determinants. For STs and most virulence and antimicrobial resistance determinants SRST2 v0.1.7^20^ was employed. For low-divergent genomic targets where a reference was available, sequences were generated using a pipeline that fed the sorted bam alignment file from SRST2 to the FreeBayes v0.9.21^21^ variant caller and then the vcf-consensus program provided by VCFtools v0.1.12b^22^. For extracting highly polymorphic or mosaic regions, the adapter trimming tool Cutadapt v1.8.3^23^, the VelvetOptimiser v2.2.5^24^ assembler, the Prodigal v2.60^25^ gene predictor and BLAST v2.2.29^26^ were employed.

### Whole genome (long read) sequencing

DNA libraries were prepared for sequencing six representative *emm82/*ST36 strains (5 invasive, one non-sterile site) of the total 15 available through ABCs), one ABCs *emm12/*ST36 strain positive for *ermB* and the *speA,H,I* genes, the single *emm-*deletion ST36 in our collection, and one invasive *emm82/*ST344 strain (sTable 3). We followed the standard PacBio Microbial Multiplexing procedure (Pacific Biosciences, Menlo Park, CA). Genomic DNA was extracted as described above for short read sequencing. Libraries were generated with the SMRTbell Express Template Prep Kit 2.0 according to the manufacturer’s suggested protocol. The libraries were then size selected on the Blue Pippin (Sage Science, Beverly, MA) to remove small DNA (< 5 kb). The final size selected libraries were sequenced for 15 h after 30 min pre-extension times on the Sequel II (Pacific Biosciences).

### De-novo genome assembly for generating single contig sequences

PacBio reads were de novo assembled using Flye v2.8. Assembled and circularized contigs were validated using BLAST + /2.6.0. Assembled sequences were then mapped and aligned using minimap (v2.170 and Samtools v1.9. Resulting assembled genomes were polished using PacBio Cromwell workflow engine^27,28^. Metrics for assemblies based upon PacBio reads and GenBank accessions for the assemblies are included in sTable 3.

### Alignment of *emm* locus regions

Prokka version 1.14.5 was used to annotate open reading frames^29^. Corresponding genomic sequences containing *emm* and flanked at the ends by the complete *sof* and the *dppB* structural genes were extracted from genomic sequences of 6 *emm82/*ST36 strains, representative strains of the recipient clonal complex (*emm12/*ST36), and two potential *emm82* donor strains. The annotated sequences were analyzed by BLAST and the regions were aligned into figures using EasyFig 2.2.3^30^.

### Phylogenetic analysis

Core genomic maximum parsimony genomic trees were generated from short read bacterial genome sequences employing kSNP3.0^31^ with a kmer size of 19. The core.tre file generated from kSNP3.0 was used by the Mega7 program to generate a phylogenetic diagram^32^.

### Alignment of single contig genomes

Progressive Mauve was used to align 8 annotated single contig PacBio genomic sequences and create an alignment diagram as described^33^.

### Determination of recombination regions within progeny

Consensus 1,774,678 bp core genomes and their alignment were derived from 4 pacbio-generated single contig genomic sequences (2 recombinant progeny strains and two recipient lineage strains) using Prokka and Progressive Mauve. This core alignment was subjected to Gubbins analysis^34^ for detection of recombinant regions. The same process was repeated to generate consensus 1,662,256 bp core genomes, derived from the same 4 single contig sequences with the addition of short read genomes from the two additional recipient lineage *emm12* strains 20,197,993 and 20,197,067 that showed the least distance from the progeny strains.

#### Opacity factor testing

Serum opacity factor (SOF) determination was performed with bacterial supernatants from specific isolates as previously described^35^.

## Results

### Potential *emm* gene switch strains in ABCs

We found 16 instances from ABCs isolates characterized during 2015–2020 (partial 2020) where strains of different *emm* types shared the same multilocus sequence types (STs) (Table 1). Since individual *emm* types are usually restricted to one ST-defined lineage, such results could reflect recent evidence of horizontal transfer of *emm* genes between unrelated strains. In 11 of these 16 instances there is clear evidence contraindicating horizontal *emm* gene transfer, in that there was a change in *emm* type based upon intra-genomic recombination within *emm* or between *emm* and downstream homologous *enn* genes. For example, within ST15, *emm161* is a 64 codon in-frame deletion derivative of *emm3*, while within ST53, type *emm164* is a hybrid gene consisting of a 5’ *emm60-*derived sequence fused to downstream *enn* gene sequence. There were only 5 clear examples of *emm* type switch events, based upon observing potential donor and progeny strains with unrelated *emm* types yet sharing the same ST. Based upon data posted at https://pubmlst.org/organisms/streptococcus-pyogenes, 4 of these examples involve switch events from at least 20 years ago (data not shown). One of the 5 *emm* switch variants was unique in that it revealed a common pattern E *emm* type (*emm82*) recently found within a classical pattern A and opacity factor negative (SOF*-*negative) major lineage, ST36. Until recently^14^, ST36 had only been associated with SOF negative *emm12* strains dating back to original Lancefield M type 12 GAS strains^36^ and type *emm12* has never been reported with STs unrelated to ST36. Additionally, *emm82* in the United States was solely associated with ST334, T type 5, and the *sof82* sequence^10,14^.

**Table 1:** *S. pyogenes* MLST types associated with multiple *emm* types recorded during 2015-present in ABCs from a dataset of more than 12,500 isolates. alues.

MLST	*emm* type A (SOF + or − , *emm* pattern)	No. of *emm* type A isolates	*emm* type B (SOF + or − , *emm* pattern)	No. of *emm* type B isolates	*emm* type B a deletion derivative of *emm* type A?
ST3	*33* (− ,D)	11	*43* (− ,D)	241	No
ST12	*91* (− ,D)	214	*29* (− ,D)	9	No
			*194* (− ,D)	1	Yes
ST14	95 (− ,D)	4	*108* (− ,D)	4	No
ST15	3 (− ,A − C)	467	*161* (− ,A − C)	1	Yes
ST28	1 (− ,A − C)	1816	*163* (− ,A − C)	1	Yes
			*227* (− ,A − C)	1	Yes
			*241* (− ,A − C)	1	Yes
ST36	*12* (− ,A − C)	849	*82* (+ ,E)	15	No
ST53	*60* (+ ,E)	244	*169* (+ ,E)	101	Yes
			*164* (+ ,A − C)	8	Yes
ST407	*89* (+ ,E)	14	*258* (+ ,E)	1	Yes
ST433	*49* (+ ,E)	1001	*151* (+ ,E)	41	Yes
ST624	*81* (+ ,E)	88	*164* (+ ,E)	1	Yes
ST853	*83* (− ,D)	309	*245* (− ,E)	1	Yes
ST1179	*18* (− ,A − C)	1	*30* (− ,D)	1	No

*emm* type designations are in italics.

Type *emm12* isolates differ from all other known pattern A-C strains in uniformly carrying a conserved inactive full length *sof12* structural gene with a single base deletion at position 2145^10,36^. All 16 *emm82/*ST36 progeny of an *emm12/*ST36 (recipient) and *emm82* (donor) strain lacked this deletion and were phenotypically SOF positive as confirmed by positive SOF tests. This correlated with the presence of typical automated bioinformatics pipeline features from the *emm82/*ST36 progeny characteristic of *emm12/*ST36, but absent from 686 *emm82* isolates recovered during 2015–2021 (Table 2). Pattern E type *emm82* was a common cause of invasive disease in ABCs during 2015–2021, and other than the 15 *emm82/*ST36 isolates collected through ABCs, consisted entirely of the ST334 clonal complex. Type *emm82/*ST334 uniformly lacked T12, the upregulated promoter (Pnga3) of the *nga-slo* virulence operon, and the active form of the extracellular *nga* product NADase (G330) (Table 2). Instead of the conserved and inactive *sof12* structural gene, *emm82/*ST334 strains carry the active *sof82* gene^10^. Certain prophage-associated exotoxin genes (*speA*, *speL,* and *speM*) and macrolide-resistance determinants were more commonly found among *emm82/*ST36 progeny than either of the parental (potential *emm82/*ST334 donor and *emm12/*ST36 recipient) strains (Table 2).

**Table 2:** Selected (CDC automated pipeline) genomic features in *emm82/*ST36, *emm12*/ST36 and *emm82/*ST334 isolates recovered during 2015–2021 from ABCs.

	T12	*emm-*like genes		Fibronectin-binding protein genes			*sda*	P*nga*-*slo* upregulated promoter	Active extracellular NADase (G330)	*drs*	Prophage-borne virulence determinants			Resistance determinants		
		*mrp*	*enn*	*sof12* indel	*Active* Sof	*fbpA*					*speA*	*speK/slaA*	*speL/M*	*ermB*	*mef*(A)	*tetO*
*emm12/*ST36 n = 666	666	0	0	666	0	0	599	666	666	619	2	3	0	10	18	0
*emm82/ST334* n = 686	0	686	680	0	686 (Sof82)	686	0	0	0	0	0	0	2	3	0	0
*emm82/ST36* n = 16	16	16	2	0	16 (Sof12)	16	11	16	16	0	6	1	5	10	1	1

### The 16 *emm82/*ST36 isolates comprise a sublineage of ST36 that arose from a double crossover “M serotype switch” event with an *emm* pattern E strain

**Figure 1:**
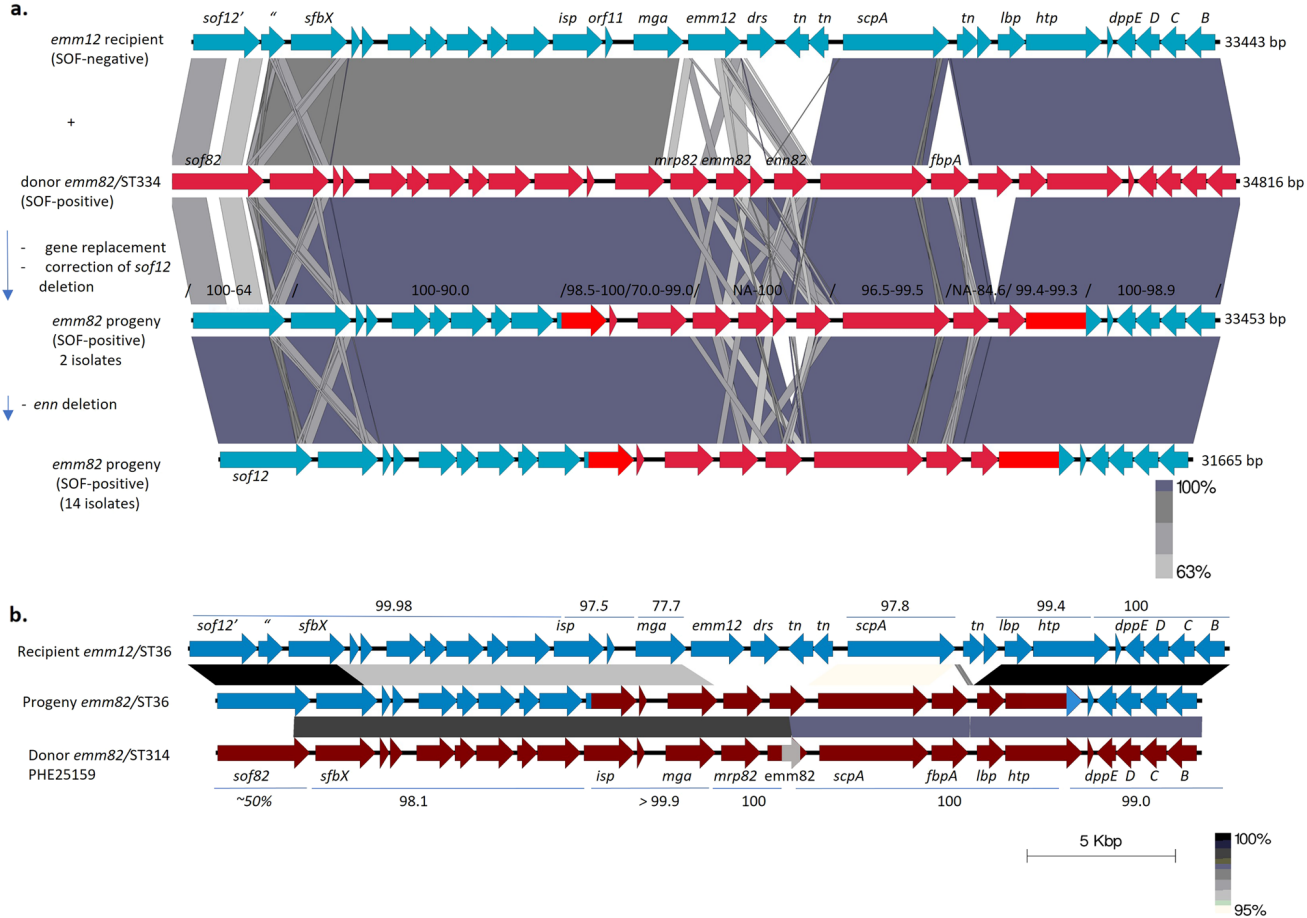
(**a**) Diagram of predicted recombination event resulting in an *emm* gene switch between an *emm12/*ST36 recipient and an *emm82 emm* gene donor strain. This genomic region is depicted from the *emm12/*ST36 strain with closest genetic relatedness to the *emm82/*ST36 cluster (isolate 20,197,993 shown in Fig. 2). The blue open reading frames are from the *emm12* recipient that carries an inactive *sof12* structural gene due to a single deleted base at position 2145 relative to the 14 *emm82* progeny strains resulting in the truncated *sof12’* and *sof12″* open reading frames. Predicted crossover points within *isp* and *htp* resulted in the replacement of this entire 17.7 kb *isp-htp* pattern A-C *emm12* region to a pattern E *emm82* region. This double crossover event resulted in the transfer of *emm-*like genes (*mrp82* and *enn82*) and fibronectin binding protein gene *fbpA* from the genetic donor (region from *emm82/*ST334 strain 20,154,608 depicted in donor strain) to replace *emm12* and *drs* (distantly related *sic* gene) within the ST36 background. In addition, the disrupted *sof12*, highly conserved within the *emm12/*ST36 background, was converted to an active *sof12* in the progeny due to the insertion of a single base at position 2145. The initial progeny (2 strains) carry the *enn82* gene which is absent within the remaining 14 progeny strains. This deletion event is predicted to have occurred through recombination between the near-identical *emm82* and *enn82* 3’ regions. The light gray crosses between *sof* and *sfbX* open reading frames depict conserved fibronectin-repeat and wall attachment motif regions. Similarly, light regions connecting *emm* and *enn-*like genes depict conserved 5’ and 3’ regions. (**b**) Same predicted recombination event as in Fig. 1a, employing *emm82/*ST314 strain PHE25159^37^, which of available potential donor type *emm82* strain genome sequences, has the highest flanking sequence homology to the progeny flanking the double crossover points. Numbers above and below depict percent identities over areas shown of the progeny to recipient and donor, respectively.

We aligned the *sof-emm-dppEDCB* region from the 16 *emm82/*ST36 isolates together with a representative putative genetic recipient *emm12/*ST36 strain and a potential genetic donor *emm82/*ST334 (Fig. 1a). This entire 31,665 bp region represented by 14 isolates shared > 99.9% sequence identity between all 16 *emm82/*ST36 isolates. The two identical crossover points (within 5’ *isp* and 3’ *htp* sequences) were evident within all 16 progeny where they differed in sequence identity from the putative *emm12/*ST36 recipient strain , revealing the replacement of a 17.6 kb fragment containing the *isp* (secreted immunogenic protein), *mga* (multiple gene activator), *emm12, drs* (distantly related serum inhibitor of complement), *scpA* (C5A peptidase), *lbp* (laminin binding protein) and *htp* (histidine triad nucleotide binding protein) with the equivalent fragment from an *emm82* strain.

**Figure 2:**
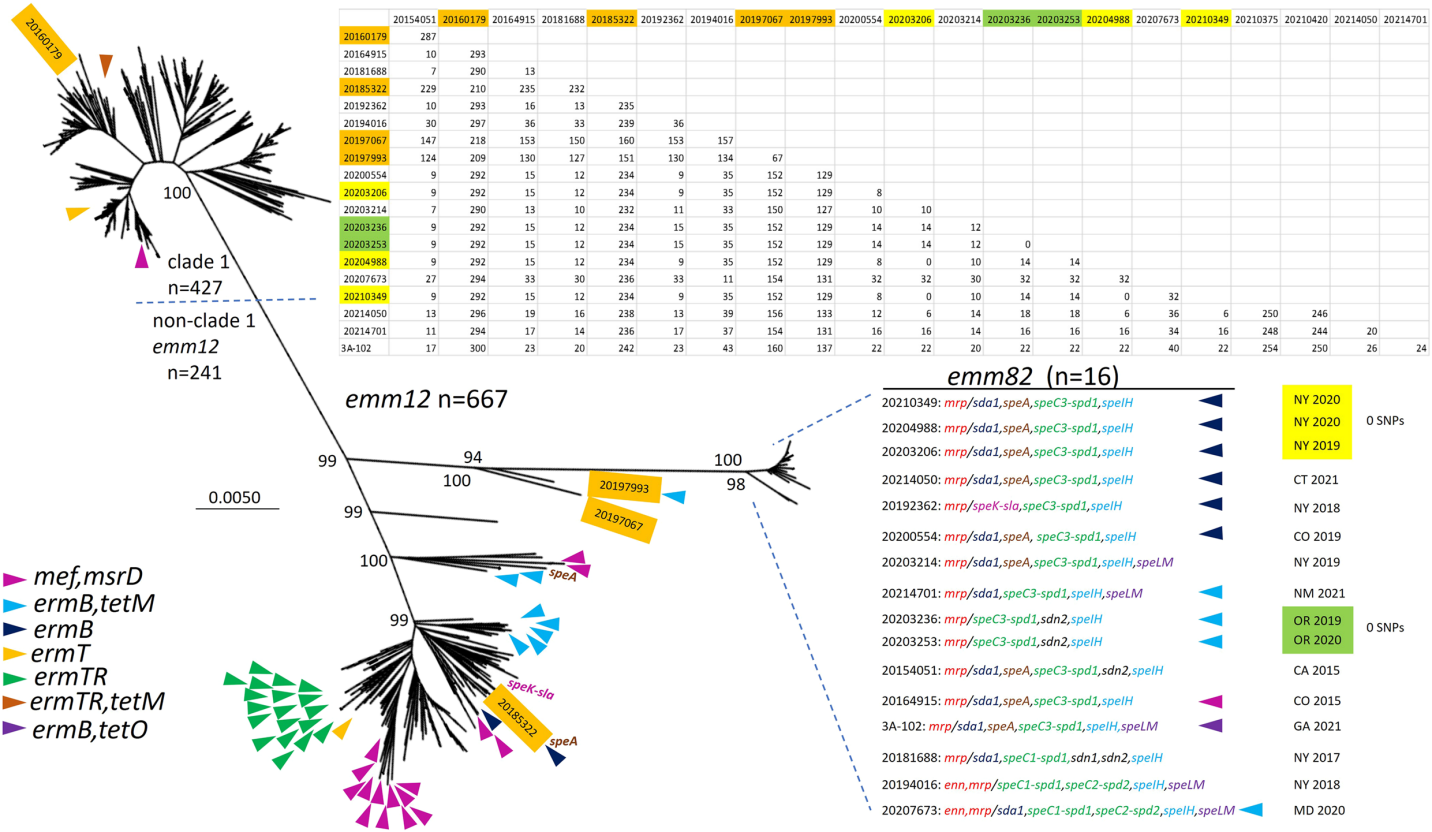
Core genomic maximum parsimony phylogenetic tree derived from short read genomes of 684 ST36 isolates including 667 invasive *emm12* isolates, a single *emm-*deletion strain (20,160,179) and 16 isolates of the *emm82 emm* switch variant sublineage. Trees were generated from short read bacterial genome sequences employing kSNP3.0^24^ with a kmer size of 19. The analysis involved 684 nucleotide sequences with a total of 5103 positions in the final dataset. Above right shows pairwise distance between the 16 *emm82/*ST36 isolates and 4 recipient lineage strains (shaded orange within the phylogram and the pairwise comparison). Two indistinguishable clusters accounting for 5 progeny isolates are highlighted in yellow and green in the phylogram and the SNP matrix. Isolates were recovered during 2015–2020 except for year 2021 *emm82/*ST36 strains 20,214,701, 20,214,050, 3A-102 and 3 *emm12* strains recovered during 2007 and 2011. Each instance of the indicated resistance genes is indicated by color-coded arrowhead in the approximate region of the phylogram. The *emm82* isolates are listed from their small branch with *emm-*family genes shown in red font, followed by prophage-encoded virulence factors, resistance gene indicators, state of isolation, and year of isolation. Hyphens indicate linkage of virulence or resistance determinants on the same accessory element. Positions of the 12 macrolide-resistant *emm82* isolates and the 50 macrolide-resistant *emm12* isolates are indicated by different arrowheads (47 clade 2 and 3 clade 1). The positions of the single *emm12* isolate carrying the linked *speK* and *slaA* genes, and the two *emm12* isolates carrying *speA* are indicated. Positions and lab identifiers of the *emm-*negative clade 1 strain 20,160,179, and three non-clade 1 *emm12* trains are indicated.

Two of the 16 *emm82/*ST36 isolates appeared to represent the progenitor progeny recombinant, in that immediately downstream of *emm82* they carried a small open reading frame (orf) and the *enn82* gene that shared sequence identity to potential *emm82* genetic donors (Fig. 1a), and this is in agreement with the depiction of these 2 isolates (20,194,016 and 20,207,673) on a branch that diverges at a closer point from the *emm12*/ST36 strains than the 14 *enn-*negative *emm82* strains (Fig. 2). The remaining 14 *emm82/*ST36 strains lacked these two orfs apparently due to a precise homologous excision event of 1788 bp between the 3’ ends of *emm82* and *enn82* that replaced the *emm82* 3’ region with the nearly identical *enn82* 3’ region (1 base difference), re-constructing an *emm82* allele with no changes in amino acid sequence. The common *emm82* and *enn82* alleles are divergent over their first 1000 bp, however these two genes share 104/105 identical bases over their last 35 codons.

The *emm82* encompassing fragment (*isp-htp* region) from the 16 recombinant progeny (Fig. 1a), shared high similarity when compared with a selection of 686 *emm82/*ST334 isolates within ABCs surveillance and *emm82* strains represented within the NCBI database (Genbank accessions LS48330 and CP007561). The contiguous 4555 bp *mrp82-emm82-enn82* region and other sections of the donated fragment shared complete sequence identity between 20,154,068 (*emm82/*ST334) and the two progenitor progeny (Fig. 1). There was small, albeit significant, sequence variation between 20,154,068 and progeny strains within other sections of the donor fragment (also shown in Fig. 1a), which prevents strong predictions of the *emm82* donor genomic lineage. The one marked difference was within the *fbpA* gene from the progeny which shared only about 85% sequence identity with all available *emm82/*ST334 strains . Strain PHE25159, an *emm82* strain recovered in the U.K. during 2014 and a 3 locus variant (ST314) of *emm82/*ST334 strains^37^ is probably more related to the putative donor strain since it also shared 99.9 – 100% sequence identity to progeny strains in deduced donated genes flanking the *emm82* gene (*isp, mga, mrp* on one side, identical *scpA, fbpA,* and *lbp* genes on other) [Fig. 1b]. In contrast to *emm82/*ST334 strains, this strain showed more divergence within a portion of the donated fragment in that it lacked an *enn* gene downstream of *emm82*, and the *emm82* gene itself diverged extensively at bases 622–993 compared to the identical *emm82* shared between ST334 and the *emm82/*ST36 strains.

### Genetic relationships among invasive ST36 strains

The unrooted phylogeny shown in Fig. 2 reveals that the 16 *emm82* strains, together with two *emm12* strains on the same branch, form a branch distinct from the other 666 ST36 strains depicted (665 *emm12* and 1 *emm* deletion strain). The 427 clade 1 isolates depicted differ from the closest *emm82/*ST36 strain (20,154,051) by 274–296 SNPs, while the remaining 241 *emm12* isolates in the phylogram differ from 20,154,051 by 124–254 SNPs. The actual distance between *emm82/*ST36 progeny and the *emm12/*ST36 recipient lineage is actually inflated by the *emm* switch event, since 54 of the 141 genomic SNPs that were exclusively conserved in the 15 progeny strains mapped within the recombinant region (data not shown).

When the same Fig. 2 tree file is depicted as a rooted tree (sFig. 1, simply rooted at midpoint of the phylogeny), it is indicative of three distinct clades that share the same most recent and as yet unknown intraspecies common ancestor. Despite the conserved recombinant region and lesser genetic distance between clade 3 (*emm82* and 2 *emm12* strains) and clade 2 (represented by strain 20,185,322), clades 1 and 2 appear to share a most recent common ancestor that first gave rise to clade 2.

The 16 *emm82* isolates had a maximal pairwise SNP difference of 43, and included 2 indistinguishable clusters (0 SNPs); one comprised of 2 isolates that differed by 12–35 SNPs from the other 14 *emm82*/ST36 isolates, and one comprised of 3 isolates that differed by 9–35 SNPs from the other 13 *emm82/*ST36 isolates (Fig. 2).

We performed recombinational analysis of progeny and recipient lineage strains to determine whether additional donor DNA segments outside of the double crossover region were evident within the progeny. Our initial strategy for this recombinational analysis consisted of generating 1,774,678 bp core genomes based upon single contig genomes from 2 progeny (*emm82* strains 20,200,554 and 20,192,362) and 2 strains of the recipient lineage ST36 (*emm12* strains 20,185,322 and an extremely rare *emm12-drs* deletion strain, 20,160,179). We subsequently repeated this analysis with the addition of short read sequence genomes from the two recipient lineage *emm12* strains (20,197,067 and 20,197,993) that were most related to the progeny strains with which they were situated on the same phylogenetic branch (Fig. 2). This repeat analysis, which utilized 1,662,256 bp core genomes generated from the 6 strains revealed the same data (sTable 4). Outside of the *emm* region double crossover region genes shown to be conserved between the putative *emm82* donor and *emm82*/ST36 progeny strains (Fig. 1) there were no additional potential donor sequences detected within the progeny genome (sTable 4). Results found were consistent with the data shown in Fig. 1 where there were 6 open reading frames that shared identity over all or most of their lengths between progeny strains 20,200,554 and 20,192,362, and differed compared to the 4 recipient lineage strains, which in turn also displayed complete or near identity to each other within these open reading frames (row 1–3 in sTable 4). One large predicted recombinant region encompassing capsular biosynthesis genes *hasA-hasC,* DNA replication/repair gene *recF*, DNA partitioning gene *parB*, and additional cellular function genes (corresponding to bases 1,901,236–1,925,891 of strain 20,200,554 [sTable 4]) was evident only within 20,160,179 relative to the other 5 ST36 strains (rows 4–6 in sTable 4). This broad recombinant region serves at least part of the basis of the genetic distance between the major *emm12* clade 1 and non clade 1 ST36 strains (Fig. 2), since the corresponding region from strains 20,160,179 and 20,185,322 is conserved with all other clade 1 strains and non-clade 1 strains, respectively (data not shown).

### The 16 *emm82/*ST36 strains share high core genome relatedness, but exhibit marked prophage and resistance element diversity

Within the set of 16 *emm82/*ST36 isolates there were 11 different complements of virulence factor genes or antimicrobial resistance genes carried on accessory elements (Fig. 2). These included 7 different exotoxin genes (*speA, speC, speH, speI, speK, speL, speM*), three deoxyribonuclease genes (*sda1, spd, sdn*), and the *slaA* gene that encodes an extracellular phospholipase. Of these, only *speC, spd,* and *sdn* were represented by multiple (2 or 3) alleles (note that strains 20,194,016 and 20,207,673 each carried 2 alleles of *speC* and *spd*). Twelve of the 16 *emm82/*ST36 isolates were macrolide resistant, including a single isolate carrying the efflux genes *mef*(A)*/msrD*, and 11 isolates with *ermB-*conferred constitutive co-resistance to macrolides and clindamycin. Seven of these *ermB-*positive isolates were resistant to tetracycline due to a neighboring *tetM* or *tetO* genes.

There were major differences from *emm82/*ST36 strains in accessory element frequencies (Table 2). Only 10 of the 667 *emm12* isolates sampled carried *ermB* or *mef*(A) determinants. There were no *emm12* isolates positive for the *speL* and *speM* genes compared to 5 of the 16 *emm82/*ST36 isolates. Only 3 of the 667 *emm12* isolates were *speA-*positive compared to 9/16 *emm82/*ST36 strains (Fig. 2).

Notably, non-clade 1 *emm12*, although accounting for only 225 of the 667 *emm12* isolate sampling, accounted for 47 of the 50 macrolide-resistant *emm12/*ST36 isolates (Fig. 2). In addition, both of the *speA-*positive *emm12* strains were within clade 2. There was a single progeny strain and 3 non-clade 1 *emm12* strains positive for the linked *speK* and *slaA* genes.

**Figure 3:**
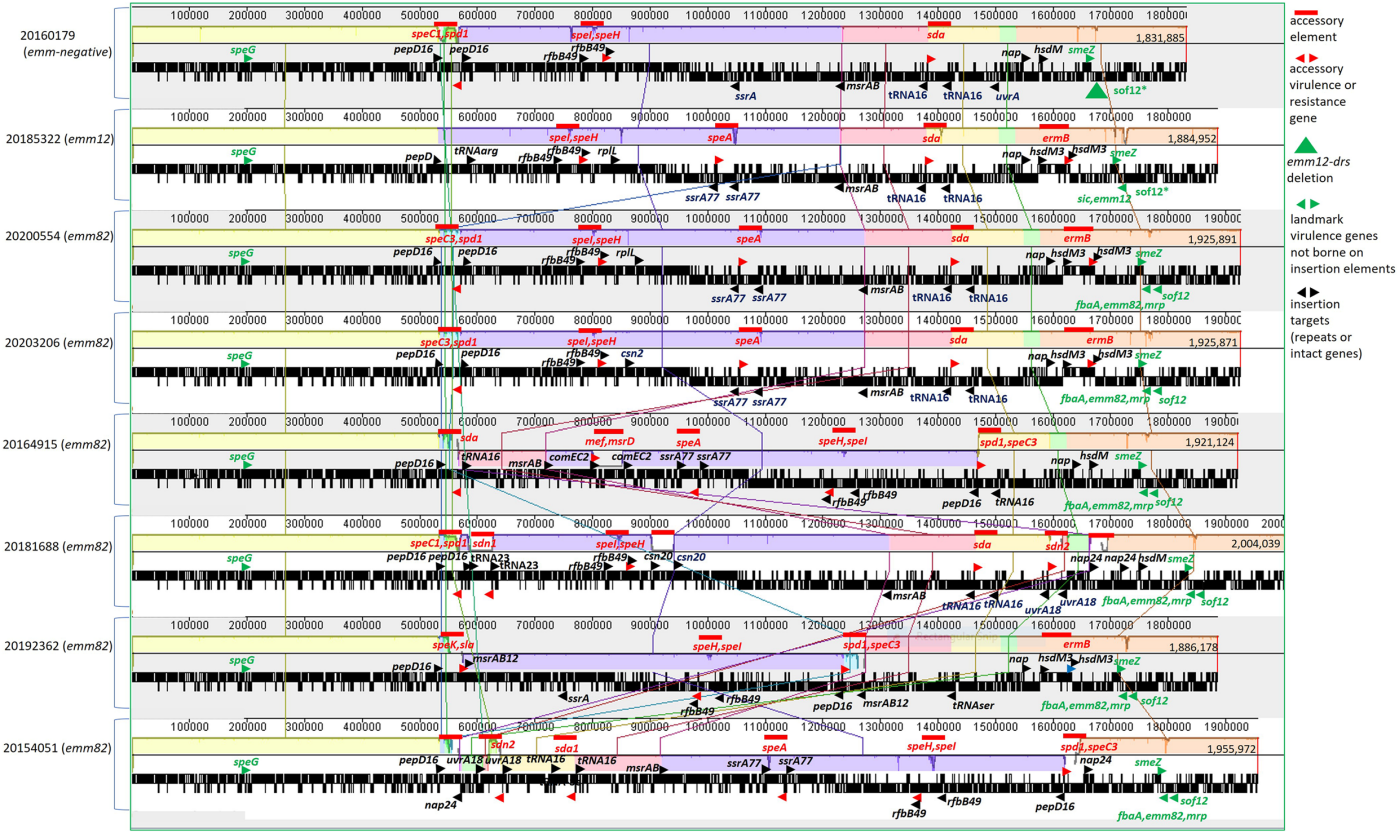
Alignment of single contig genomes from recipient ST36 lineage and *emm82*/ST36 progeny strains. Each genome starts at the consensus site just upstream of the chromosomal replication initiator protein gene *dnaA*. Landmark virulence genes not associated with accessory elements are indicated in green font with indicated orientations noted for forward (top) and reverse (bottom) strands. Tandem repeated sequences flanking prophages or insertion elements are also indicated with number of bases and are listed below. The positions of uninterrupted genes corresponding to repeat sequences are indicated within some strains (tRNA23 corresponds to *tRNA-arg* in strain 20,185,322 and tRNA16 corresponds to *tRNA-ser* in strain 20,192,362). Accessory elements (Prophages or resistance elements) are indicated in red with relative orientations. The sof* designation refers to conserved inactive single base deletion *sof12* derivative. Multiple genes separated by commas and are oriented with the bottom or top strand in direction and order of gene transcription. Conserved genomic sections are color coded. Major inverted genomic sections are indicated for strains 20,164,915, 20,192,362, and 20,154,051 relative to the 5 other strains in the alignment. Genome sizes are indicated at right end of each genome.Flanking repeat sequences and corresponding genes (alphabetical order): ***comEC2*:** GG, within ***comEC*** competence family protein gene. ***csn20*:** GCTATGCTGTTTTGAATGGT, downstream of CRISPR-associated protein gene ***csn2***. ***hsdM3*:** GGG, within type I restriction/modification system protein gene ***hsdM***. ***msrAB12*:**TATTATATCAGA, downstream of peptide methionine sulfoxide reductase genes ***msrA-msrB***. ***nap24*:** TATGATGAACATGCAAAACATGAT, overlaps 5’ end (start codon underlined) of nucleoid-associated protein gene ***nap***. ***pepD16*:** CATGTACAACTATACT, intragenic within ***pepD*** dipeptidase gene. ***rfb49*:** AAACTCAAGAAGTGATTAAATAAAACATTAAACAACCTTGTCATATCAA, 3′ 23 bases of ***rfbB*/*rmlB*** (dATP-glucose-4,6-dehydratase) and 26 bases of *rfbB*-*mutT* intergenic region. ***ssrA77*:**ATGCTTACCGTAAGTAATCATAACTTACT**AAAACCTTG**TTACAT**CAAGGTTTT**TTCTTTTTGTCTTGTTCATGAGTTencompassing one of 2 transcriptional terminators downstream of ***ssrA*** (tmRNA gene). ***tRNA16*:** AGGAGAGGAGGGGATT, overlaps exactly with 5’ end of ***tRNA-ser*** gene. ***tRNA23*:** GATTCCGGCAGGGGTCATTATTG, encompasses 3’ end of ***tRNA-arg*** gene. ***uvrA18*:** CTTATATTATAACAAAAA, downstream of excinuclease ABC subunit A protein gene ***uvrA***.

Among the 6 *emm82/*ST36 strains for which single contig sequences were obtained, the number of prophages ranged from 3 (strain 20,192,362) to 7 (strain 20,181,688) [Fig. 3.]. Within certain prophages that shared the same insertion site, virulence features, and conserved flanking sequences, considerable genetic variation was apparent, likely indicative of frequent inter- and intra- phage recombination events. For example, there were 3 different *speA-*harboring prophage derivatives among the 4 *speA-*positive *emm82/*ST36 strains with single contig genomic sequences (sFig. 2). Three prophages (2 in 20,181,688 and 1 in 20,154,051) did not carry known virulence determinants (Fig. 3).

All 16 *emm82/*ST36 strains carried prophages harboring *speC/spd* and *speI/speH*. Of 8 *speC/spd—*containing prophages compared from *emm82* progeny, only two (20,200,554 and 20,203,206) shared an entirely conserved organization and high homology (sFig. 3). All 16 *emm82/*ST36 progeny contained a highly conserved prophage harboring the linked *speI/speH* genes inserted within a conserved genomic site and flanked by the same 49 bp tandem repeat (Fig. 3). Eleven of the 16 *emm82/*ST36 strains carried the prophage-borne *sda1* (corresponding to *sdaD2* in GenBank accession CP000261) gene. Each of the 7 prophages harboring *sda1* shown were inserted at the same *tRNA-Ser* gene, and with the exception of 20,164,915 exhibited flanking repeats corresponding to the 3′ 16 bases of the *tRNA-Ser* gene (Fig. 3). These prophages, with the exception of the *sda1*-harboring prophage from 20,164,915, were highly similar to each other and to a prophage from reference M12 strain MGAS9429 (NCBI accession CP000259) [sFig. 4].

### Varied genomic organization patterns of emm82/ST36 strains coincide with recombination between prophages

Among 8 single contig genomic sequences (Fig. 3), the *emm82*/ST36 strains 20,200,554, 20,203,206, and 20,181,668 share the overall genomic organization of the *emm12* strain 20,185,322 and clade 1 *emm-*negative strain 20,160,179 (Fig. 3). In contrast, three of the *emm82/*ST36 strains (20,164,915, 20,192,362, and 20,154,051) revealed massive genomic inversions ranging from approximately 700 kb (20,192,362) to 1100 kb (20,154,051), while their pairwise differences compared to non-inverted strains only ranged from 7–13 SNPs. While there are opposite orientations of core genomic segments, they are syntenic and differ only by accessory element content (Fig. 3). Each of these 3 genomic inversions appeared to have been triggered due to recombination between 2 different phages, as described previously for M serotype 3 strains^38^. In each of the 3 genomic inversion strains, the oriC-proximal genomic boundary of the inversion is flanked by a phage that is inserted immediately after bases 1–20 of the *pepD* gene, with *pepD* bases 4–20 repeated at the other end of the genomic inversion and adjacent to a distinct prophage (Fig. 3, only *emm12* strain 20,185,322 lacks a prophage within *pepD*).

**Figure 4:**
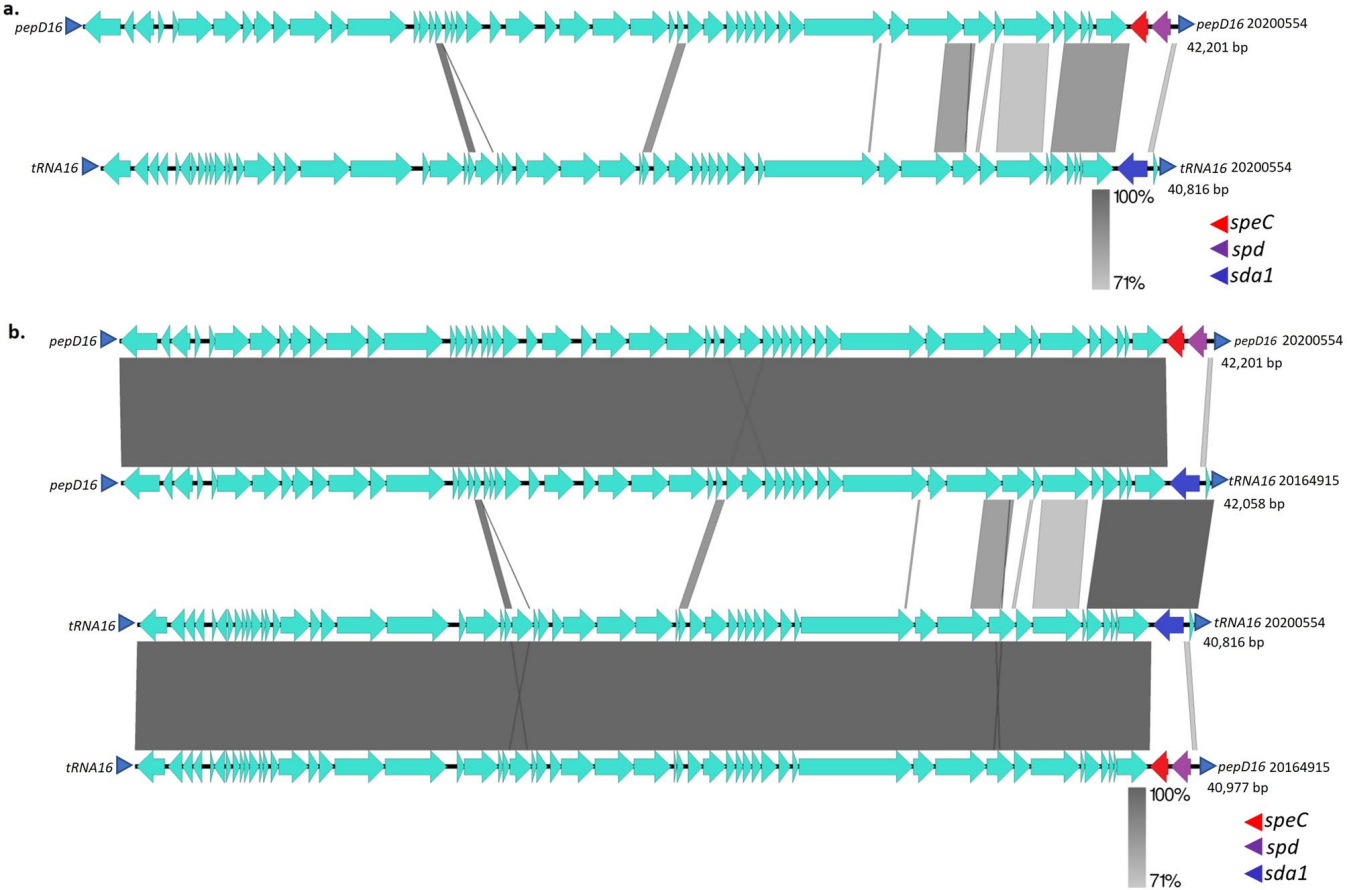
(**a**) Alignment of prophages carrying *speC/spd* and *sda* from strain 20,200,554 The small blue arrowhead flanking each prophage indicate repeat sequences despicted in Fig. 3 and described in the Fig. 3 legend. (**b**) Alignment of prophages carrying *speC/spd* and *sda* from strains 20,200,554 and 20,164,915.

There is considerable homology between different prophages within virulence determinant regions^38^, as shown for the two strain 20,200,554 prophages inserted within the *pepD* and the *tRNA-*Ser genes (Fig. 4a). This relates to the genomic inversion evident in strain 20,164,915 relative to strain 20,200,554 (Fig. 3). Strain 20,164,915, instead of displaying the repeated *pepD* sequence (*pepD16*) flanking its prophage carrying *speC* and *spd* as in strain 20,200,554, only has the *pepD16* sequence adjacent to the genome oriC—proximal end of the prophage (Figs. 3, 4a). At the other end of this prophage is the *tRNA16* repeat that flanks both ends of prophage 20200554*sda1*. The hybrid nature of both prophages from strain 20,164,915 relative to strain 20,200,554 is evident (Fig. 4b) and coincides with the large genomic inversion between the two prophages that each are flanked by *pepD16* and *tRNA16* (Fig. 3). The single phage carrying *speK/sla* (in strain 20,192,362) also shared extensive homology with the 20,200,554 *speC/spd* phage (sFig. 5), likely to be a product of an intra-genomic recombination/inversion event involving prophages inserted within *pepD* and *msrB*. Finally, the genomic inversion within strain 20,154,051 likely involved prophages mapping within the *pepD* and *nap* genes (sFig. 6) separated by approximately 1000 kb (uninterrupted *nap* gene evident within 5 strains lacking large inversions in Fig. 3). To summarize, the *emm82* strains 20,200,554, 20,203,206, and 20,181,688 shared the same genomic insertion sites for their *speC/spd* and *sda1*- containing phages (within *pepD* and tRNA-ser genes, respectively), while strains 20,164,915 and 20,154,051 each revealed hybrid sets of repeats flanking these prophages that coincided with the endpoints of their genomic inversions. Strain 20,192,362 appears to have undergone yet another recombination event driven by prophages harboring *speK/slaA* and *spd/speC* genes, shown by shared phage homologies and hybrid sets of repeats (*pepD16* and *msrAB12*) flanking both prophages (Figs. 3 and sFig. 7).

#### Resistance elements

**Figure 5:**
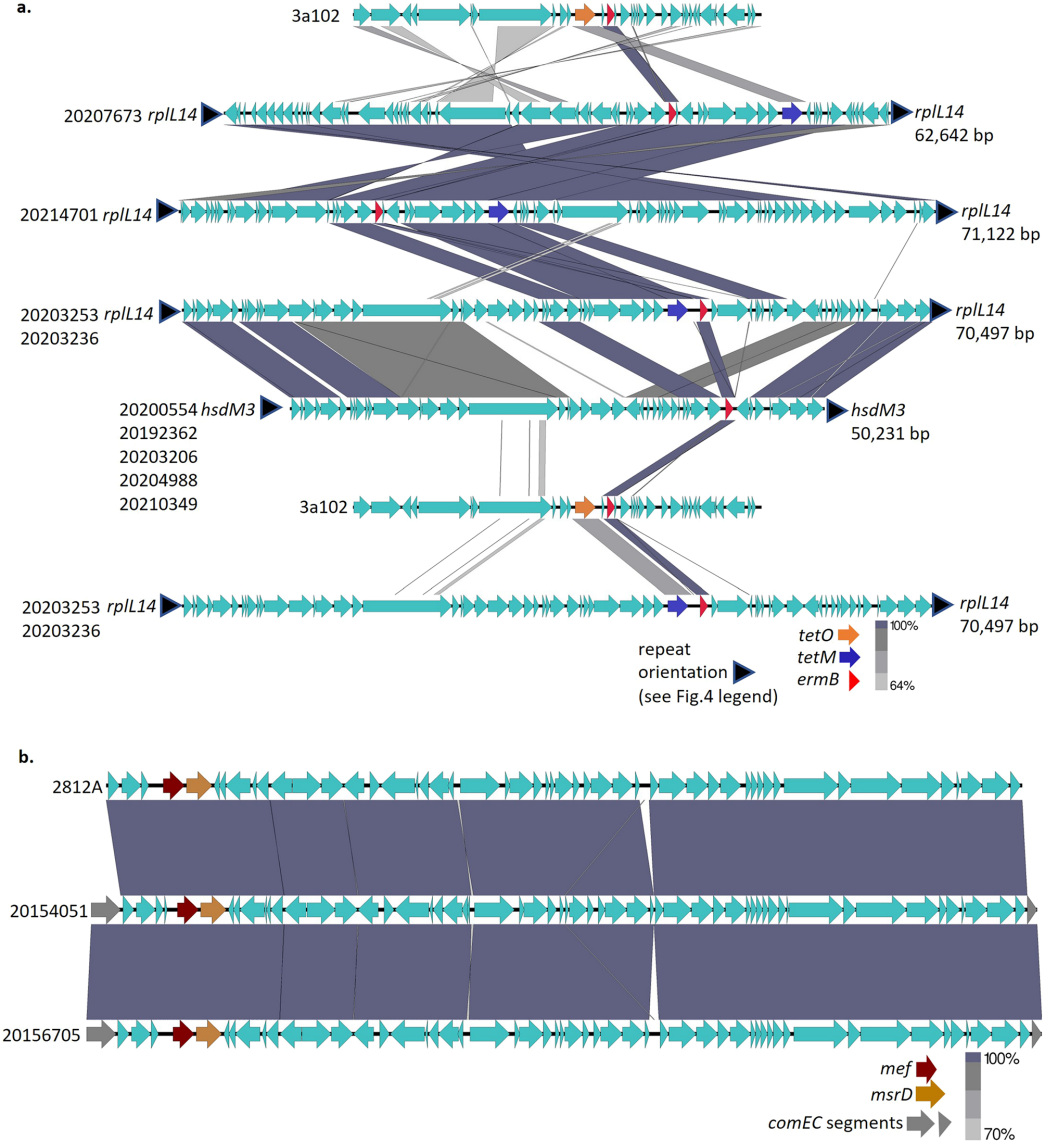
(**a**) Transposons harboring *ermB* in *emm82/*ST36 strains. A partial element carrying *ermB* and *tetO* was extracted from pharyngitis strain 3a102. (**b**) Element harboring composite prophage/transposon ø*Tn1207* in strains 20,154,051(*emm82/*ST36) and 20,156,705(*emm12/*ST36) resistant to erythromycin and susceptible to clindamycin. The upper sequence depiction is derived from strain 2812A described in reference 40.

In addition to a broad diversity of prophage content, 12 of the 16 *emm82/*ST36 strains harbored accessory elements harboring macrolide resistance determinants (Figs. 2, 5a,b). Six *emm82/*ST36 isolates (pairwise genomic distance ranging from 0 to 16 SNPs and shown in Fig. 2 phylogram), and the clade 2 *emm12* strain 20,185,322, carried an identical *ermB-*harboring element inserted within a structural gene encoding a type I restriction-modification component (designated *hsdM* in Fig. 3) [Fig. 5a]. This element is highly similar to the element recently described from strain GAS4764HUB^39^. Three related elements harboring both *ermB* and *tetM* were found among 4 *emm82/*ST36 isolates that had a pairwise genomic distance ranging from 0–33 SNPs. These 4 isolates shared the same insertion site for these *ermB*/*tetR* elements just downstream of the 50S ribosomal protein L7/L12 gene *rplL* (genomic location of intact gene shown in Fig. 3 for strains 20,185,322 and 20,200,554). The single *mef*(A)*-msrD*-positive *emm82/*ST36 strain 20,164,915 (genomic pairwise distance of 13–36 SNPs from 13 other *emm82/*ST36) harbored the composite prophage ø1207.3 inserted within the *comEC* gene^40^ [Fig. 5b].

Available case and demographic data for the 16 documented *emm82/*ST36.

Four of the 16 isolates (wound isolates 20,154,051, 20,207,673, and 20,214,701; pharyngitis isolate 3a102) were from non-sterile sources (Table 3) and therefore excluded from ABCs, so epidemiologic data were not available.

**Table 3:** Features associated with 16 emm82/ST36 clinical isolates.

Isolate	Year isolated	State	sex	Age	clinical specimen	Cellulitis	osteomyelitis	pneumonia	arthritis	septic shock	STSS	Experiencing homelessness	asthma	dementia	Cocaine or methamphetamine abuse	alcoholism	diabetes	Smoker
20154051^A^	2015	CA			Nonsterile (wound)													
20,164,915	2015	CO	F	20–40	Deep tissue													x
20,181,688	2017	NY	F	> 60	Blood	x							x	x				
20,194,016	2018	NY	M	40–60	Blood	x												
20,192,362	2018	NY	M	20–40	Blood													
20,200,554	2019	CO	M	20–40	Blood			x		x		x						
20,203,214	2019	NY	F	> 60	Blood								x					
20,203,206	2019	NY	F	20–40	Blood								x		x	x		
20,210,349	2020	NY	F	20–40	Blood	x									x	x		
20,204,988	2020	NY	F	20–40	Blood		x	x		x	x				x			
20,203,236	2019	OR	M	> 60	Blood									x				
20,203,253	2020	OR	M	> 60	Blood		x	x		x						x		
20,207,673	2020	MD			Nonsterile (wound)													
20,214,701	2021	NM			Nonsterile (wound)													
20,214,050	2021	CT	M	50–60	Joint												x	
3a102	2021	GA		5–18	Nonsterile (pharyngitis)													

The 12 invasive cases with available data were divided between 4 states among adults within the age range of 21–79 years. Ten of the 12 patients had certain predisposing conditions or circumstances for invasive GAS disease, including at least two of following: smoker, alcohol abuse, drug abuse, asthma, obesity, diabetes, dementia, heart disease, experiencing homelessness. These 12 isolates were inclusive of two small clusters of genomically identical isolates (0 SNPs). A three isolate cluster recovered in New York during 2019–2020 included 3 younger females who used cocaine and methamphetamines. Two of the 3 patients resided within the same county and the same zip code. A two isolate cluster was recovered during 2019–2020 from two older males residing within the same county in Oregon. Counting non-invasive isolates as well, *emm82/*ST36 strains were found within 8 different states. The twelve invasive cases were associated with multiple clinical associations, ranging from bacteremia without focus to streptococcal toxic shock syndrome (Table 3).

## Discussion

M serotype 12 (*emm12*) has been a major cause of both invasive and non-invasive infections within the United States for decades (https://www.cdc.gov/abcs/bact-facts-interactive-dashboard.html, 3). All known *emm12* strains characterized, dating back to 70 year old reference strains, have been *emm* pattern A, ST36, and carry a conserved inactive *sof* gene^6,10,36^. M serotype 82, formerly provisional M serotype PT180^10,41^ was not among the most frequent causes of invasive GAS disease during 1997–2014, while *emm12* was among the 4 most common invasive types during each of these 18 years. Since 2015 there has been a marked increase in the proportion of *emm82* invasive GAS where it has become one of the predominant *emm* types. Remarkably, during each of the full years 2020 and 2021, *emm82* was the second most prevalent *emm* type in ABCs, while *emm12* was not among the most prevalent 6 *emm* types (see https://www.cdc.gov/abcs/reports-findings/surv-reports.html for surveillance reports since 1997). During this period, all *emm82* were of the ST334 clonal complex, with the exception of the small number of *emm82/*ST36 described in this report.

Recently there has been a resurgence of invasive GAS disease incidence, especially that caused by *emm* pattern E types such as *emm82*^11^. This overall increase has been driven by temporally highly related invasive GAS isolate clusters recovered from adults within the United States, which are detectable by whole genome sequence data^2,11,14,16^. The 3 *emm* types (*emm49, emm92,* and *emm82*) contributing the highest numbers of clustering invasive case isolates during 2015–2018 were all pattern E types^11^, and this trend of increasing pattern E invasive cases is ongoing (unpublished). Concurrently, macrolide resistance in invasive GAS has approximately doubled during the past decade, and this increase has also been driven by disease clusters^42^.

A primary goal of this study was to examine in more detail a very unusual emergent strain. From the analysis of ABCs isolates characterized since 2015 using whole genome sequencing, we found that the incidence of apparent horizontal transfer events resulting in recombinational replacement of the *emm* locus (ie, an identical *emm* type superimposed upon unrelated MLST types) is a very uncommon event. The novel pattern E *emm82/*ST36 M type-switch sublineage is unprecedented in combining a predominant cluster E M protein^12^ with a pattern A genomic lineage^6,7^. Further, the high diversity of prophages and resistance elements within this closely related set of isolates as judged by core genome relatedness is remarkable. It is possible that continued increases of pattern E *emm* type disease-causing strains such as *emm82* will lead to increased generation of such novel recombinant strains. Included within the ST36 lineage for the first time are related M-like protein genes (*mrp* and *enn*), fibronectin binding protein gene *fbpA*, and an active multifunctional *sof* gene which contribute additional host protein binding features^7–9^. The clustering tendency, with two independent sets of indistinguishable isolates, and high proportion of macrolide-resistance of the *emm82/*ST36 lineage is consistent with the high cluster/odds ratio values calculated for invasive *emm82/*ST334 strains^11^, and is also in agreement with recent increased incidence of macrolide resistance that is primarily driven by clustering pattern E strains^42^.

In addition to its serum opacification activity, Sof is an adhesin/invasin that binds to different host proteins, and in at least some strains is an antiphagocytic factor in human blood^8,43^. Pattern A-C *emm* types such as *emm1, emm3, emm5, emm6*, and *emm12* are invariably opacity factor negative due to the lack of a functional *sof* gene. In addition to lacking *sof,* pattern A strains also lack the *emm* family protein genes *mrp* and *enn.* Even though the region flanking *sof* and the cotranscribed *sfbX* (streptococcal fibronectin binding gene X) is very conserved between patterns A-C and E strains, there has been no known horizontal transfers of the *sof-sfbX* operons into pattern A-C lineages. It follows also that pattern E lineages are invariably associated with an active *sof* gene, as judged by the opacity factor phenotype^7^. This observation, combined with the correction of the defective *sof12* in an emergent *emm82/*ST36 background suggests that the fitness of pattern E type strains depends upon combined roles of *sof* and *emm* region genes. *emm12* strains appear to be very unique among pattern A-C strains that carry no remnant of the *sof12-sfbX* operons. *emm12 strains* carry full length *sof-sfbX* operons that contain a rigidly conserved single base deletion within the also highly conserved *sof12* structural gene, predicted in all strains characterized to date to encode a truncated Sof precursor protein that lacks a portion of the enzymatic domain, all fibronectin-binding repeats, and the C-terminal membrane anchor^36^.

This *emm12* to *emm82* switch event is analogous to pneumococcal capsular serotype switch events, which also result from genetic exchange of a large chromosomal region. In pneumococci such double crossover serotype switch events are often associated with multiple unlinked recombination events involving the same genetic donor^44,45^. At least in this specific example described in our report, no additional recombination events outside of the *emm82* containing donated fragments were evident within the *emm82/*ST36 progeny. There is abundant history of identical *emm* genes associated with differing ST lineages, but very few instances of different *emm* types sharing the same ST^14^. Although we showed evidence that an *emm82/*ST314 strain might have been a recent donor, recombination at this region and lack of sufficient sampling could be confounding.

In recent years *emm82/*ST334 has rapidly emerged as a major cause of invasive disease, however it is unknown how impactful the *emm82/*ST36 lineage will be. Although currently low in incidence, its relatively sudden appearance within 8 different states, association with varied complements of virulence and resistance determinants, as well as recovery from both invasive and non-invasive clinical specimens during the past few years is reason for close monitoring. At the time of receiving the journal review of our work we received an additional ABCs *emm82/*ST36 strain recovered during 2022 in New York. We became aware from previous work^37^ of an additional strain of this complex recovered in Canada sometime during 2010–2013 from unknown clinical source, and 2 strains in the U.K. during 2014 (one from invasive disease and one from scarlet fever). We found that all 4 strains are highly related to the overall U.S. *emm82/*ST36 strain set (differing by 5–18 SNPs from closest neighbors as well sharing identical and related accessory components). Importantly, this is indicative of a continuing emergence and a much wider global spread of this novel strain complex.

It is likely that detection of new and potentially dangerous strains in the U.S. is delayed by the limited ABCs surveillance scope (approximately 10% of the U.S. population within 10 states), and perhaps even more so by the lack of systematic surveillance of non-invasive GAS disease. Probably the actual recombination event creating this emergent *emm82/*ST36 strain occurred much more recently than an estimate of 56 years (as estimated by roughly 1.7 SNPs per year^46^), since it is highly likely that the recipient lineage *emm12* strain most related to the *emm82/*ST36 lineage has not been identified. 

### Supplementary Information

Supplementary Information.

Supplementary Table 2.

## Data Availability

All data incorporated in this manuscript is available as described within the.methods and other text.
