# Supplementary material for: A novel invasive Streptococcus pyogenes variant sublineage derived through recombinational replacement of the emm12 genomic region
Source: Sci Rep. 2023 Dec 6;13:21510. doi: 10.1038/s41598-023-48035-2 (PMC10700362; doi:10.1038/s41598-023-48035-2)
Supplement: Supplementary file 1 — Supplementary Information. [file 41598_2023_48035_MOESM1_ESM.docx]

**A novel invasive *Streptococcus pyogenes* variant sublineage derived through recombinational replacement of the *emm12* genomic region.**

Yvette Unoarumhi^1^, Morgan L. Davis^1^, Lori A. Rowe^1^, Saundra Mathi^2^s, Zhongya Li^2^, Sopio Chochua^2^, Yuan Li^2^, Lesley McGee^2^, Benjamin J. Metcalf^2^, Justin S. Lee^1^ & Bernard Beall*^2^

^1^Centers for Disease Control and Prevention, National Center for Emerging and Zoonotic Infectious Diseases, Division Scientific Resources, Biotechnology Core Facility Branch, Atlanta, Georgia, USA.

^2^Centers for Disease Control and Prevention, National Center for Immunization and Respiratory Diseases, Division of Bacterial Diseases, Respiratory Diseases Branch, Atlanta, Georgia, USA.

sTable 1. Assembly metrics for the Illumina-generated genomic sequences for the 684 ST36 strains described in this work

|  | range | average |
| --- | --- | --- |
| Number of contigs | 31-199 | 42 |
| longest contig (bases) | 77554-507426 | 275480 |
| N50 (bases) | 25328-225801 | 149569 |
| total bases | 1720418-1946058 | 1801218 |

| sTable 3. Metrics associated with PacBio-generated genomic sequences for study | | | | |  |  |  |  |  |  |  |  |
| --- | --- | --- | --- | --- | --- | --- | --- | --- | --- | --- | --- | --- |
| assembly | Coverage | Circular | Total read length | Number of Contigs | Total size of contig | scaffold %A | scaffold %C | scaffold %G | scaffold %T | Gaps | Accession | biosample |
| 20154608(*emm82/*ST334) | 567 | Yes | 1115661823 | 1 | 1825665 | 30.83 | 19.3 | 19.22 | 30.66 | 0 | CP118306 | SAMN33310438 |
| 20200554(*emm82/*ST36) | 201 | Yes | 413056079 | 1 | 1925893 | 30.65 | 19.44 | 18.96 | 30.95 | 0 | CP118307 | SAMN33312873 |
| 20203206(emm82/ST36) | 191 | Yes | 392336168 | 1 | 1925874 | 30.95 | 18.97 | 19.44 | 30.65 | 0 | CP118308 | SAMN33314856 |
| 20192362(emm82/ST36) | 205 | Yes | 417465471 | 1 | 1886178 | 30.82 | 19.2 | 19.18 | 30.8 | 0 | CP118309 | SAMN33314877 |
| 20154051(emm82/ST36) | 251 | Yes | 532788583 | 1 | 1955972 | 30.59 | 19.78 | 18.76 | 30.88 | 0 | CP118310 | SAMN33315611 |
| 20164915(emm82/ST36) | 286 | Yes | 582224968 | 1 | 1921129 | 30.79 | 19.03 | 19.53 | 30.65 | 0 | CP118311 | SAMN33315639 |
| 20186188(emm82/ST36) | 236 | Yes | 501641187 | 1 | 2004040 | 30.64 | 19.45 | 19.07 | 30.85 | 0 | CP118312 | SAMN33315834 |
| 20185322(emm12/ST36) | 307 | Yes | 611921074 | 1 | 1884952 | 30.7 | 19.39 | 19 | 30.91 | 0 | CP118481 | SAMN33325090 |
| 20160179(emm-negative/ST36) | 278 | Yes | 542032548 | 1 | 1831883 | 30.6 | 19.46 | 19.08 | 30.86 | 0 | CP118482 | SAMN33325377 |

sTable 4. Genomic coordinates and genes corresponding to recombination regions detected in gubbins analysis of 1,662,256 bp core genomes from two progeny (20200554 and 20192362), and 4 representative recipient lineage strains.

| Genomic Coordinates^A^ | 20200554  *emm82*/ST36  genome fragment  length | open reading frames | 20192362  *emm82*/ST36 | 20197993^C^  emm12/ST36 | 20197067^C^  *emm12*/ST36 | 20185322^C^  *emm12*/ST36 | 20160170  Clade 1  *emm* neg/ST36 |
| --- | --- | --- | --- | --- | --- | --- | --- |
| 1. 900776-904673  1768370-1772256 | 3887 bp | *Isp*^B^*, orf11*^B^*, mga*^B^ | 100% | 90.3%  99.9% | 90.4%  99.9% | 90.6%  99.7% | 90.6% |
| 2. 877476-880811  1761955-1765290 | 3336 bp | *scpA*^B^ | 100% | 98.4%  100% | 98.4%  100% | 98.4%  100% | 98.4% |
| 3. 1659203-1662231  1756949-1759977 | 3029 bp | *lbp*^B^*, htp*^B^ | 100% | 99.4%  100% | 99.4%  100% | 99.4%  100% | 99.4% |
| 4. 1031370-1035950  1901263-1905843 | 4581 bp | *ecfA1,pgsA,hyp,*  *albF,albE* | 100% | 100% | 100% | 100%  99.7% | 99.7% |
| 5. 880812-899190  1905929-1924307 | 18379 bp | *hasA-C*,*yaaA*,*recF*,*glcU,*  *guaB*,*trpS2*, *hyp*,*yheS2, hyp,* *tRNA-Asn,tRNA-Glu,tRNA-Arg,*  *rlmH, htrA* | 100% | 100% | 100% | 100%  99.4% | 99.4% |
| 6. 899191-900775  1924208-1925891 | 1585bp | *htrA,parB* | 100% | 100% | 100% | 100%  99.6% | 99.6% |

^A^ Top range corresponds to aligned core genomes of all 6 strains included in analysis. The bottom range corresponds to the homologous segment from single contig genome sequence derived from strain 20200554(*emm82/*ST36) depicted in Fig. 3.

^B^ These open reading frames are apparent within recombinational (*emm* region switch) fragment depicted in Figure 1.

^C^ Upper percentage values correspond to percent identity between corresponding genome fragment with strain 20200554 depicted in column 2. Lower percentage values correspond to percent identity over the fragment to the adjacent isolate fragment on the right. The data shown in column 6/ rows 4-6 is representative of the difference shown between each clade 1 strain and all other ST36 strains depicted in the Fig. 2 phylogram.


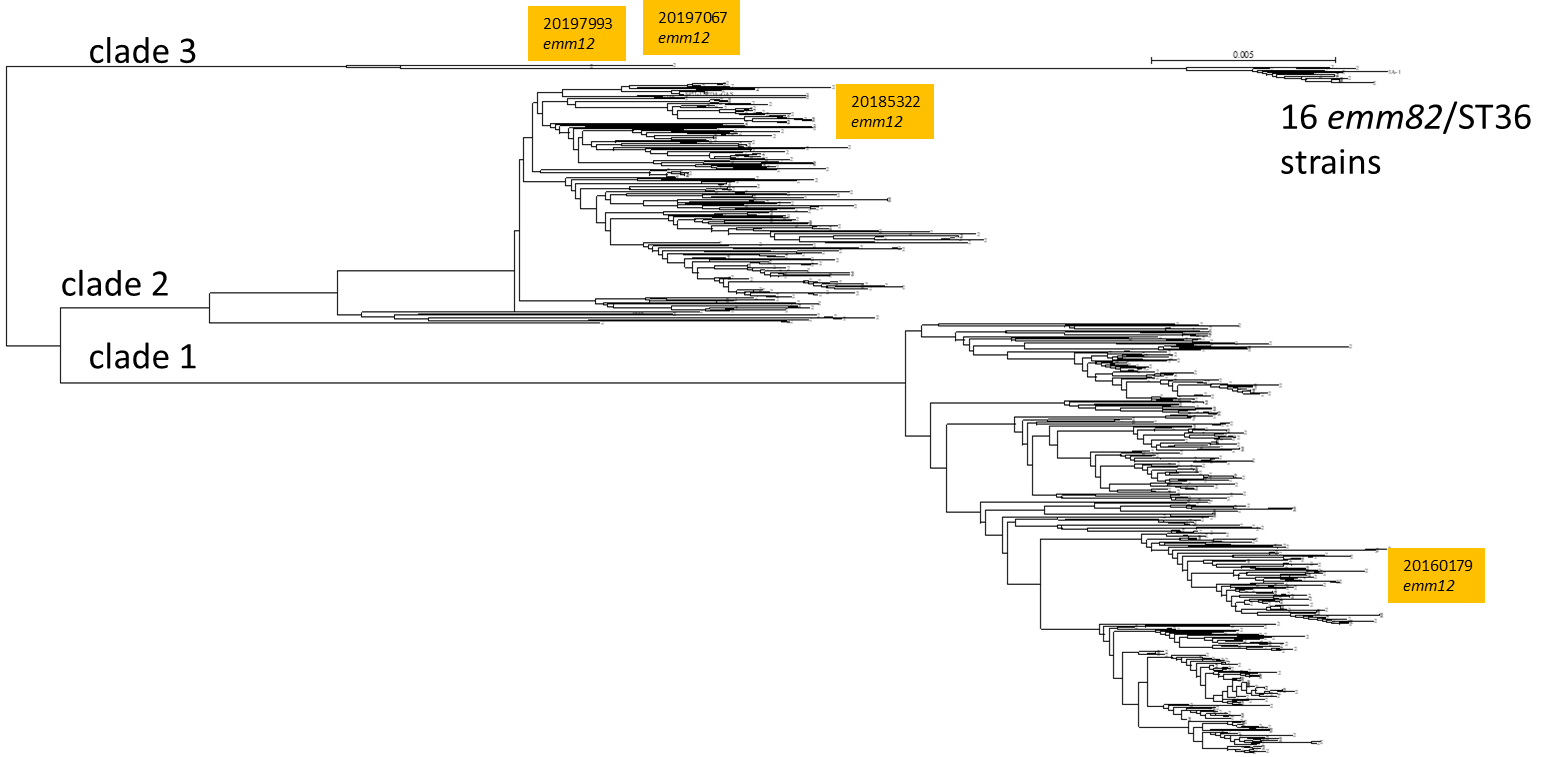


**sFigure 1.** Rooted phylogenetic tree employing same data as in unrooted tree in Fig. 2, indicative of a common ancestor that gave rise to 3 independent clades. Isolates shaded in orange were employed for gubbins (all 4, see Table 3) and for generation of single contigs (Fig. 3).


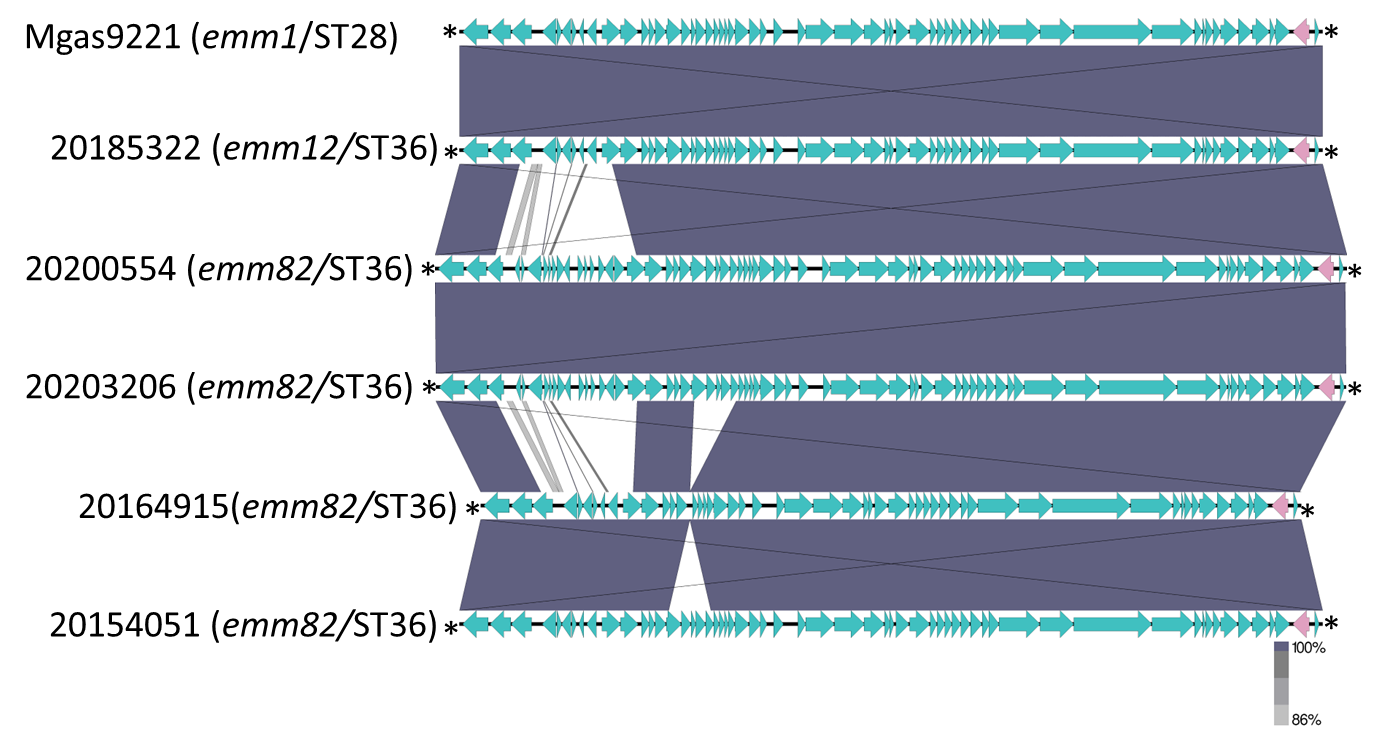


**sFigure 2.** Alignment of *speA*-containing prophage between *emm1/*ST28, *emm12/*ST36 and *emm82/*ST36 strains. Mgas2221 was derived from GenBank accession CP043530.1. The pink orf represents *speA.*

*77 bp tandem repeat


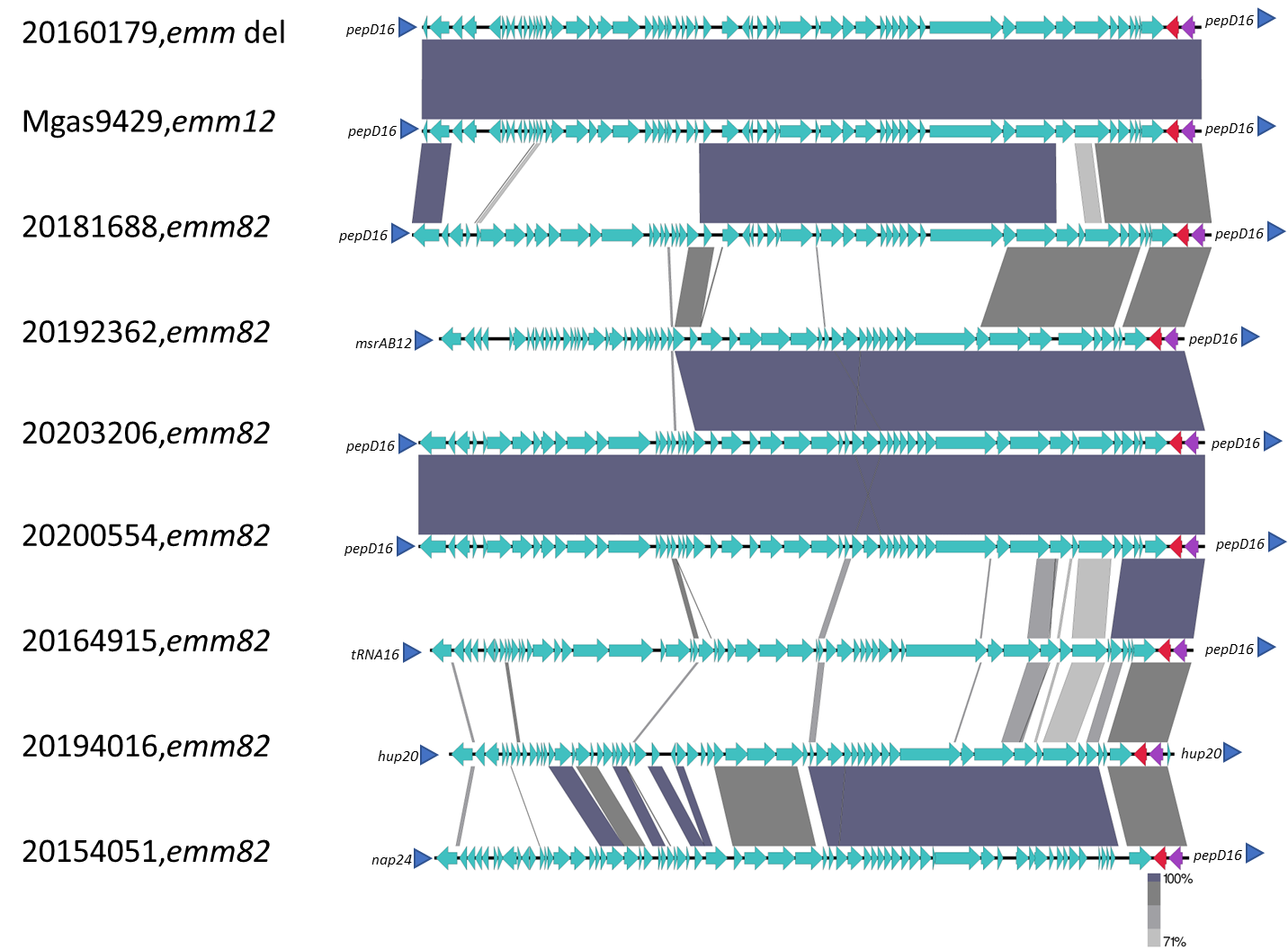
 **sFigure 3.** Alignment of prophages from the clonal complex ST36 harboring the linked *speC* (red arrow) and *spd* (purple arrow) genes. The small blue triangles represent tandem repeats described in Fig. 3 legend. Mgas9429 sequence was from NCBI reference sequence NC_008021.1.


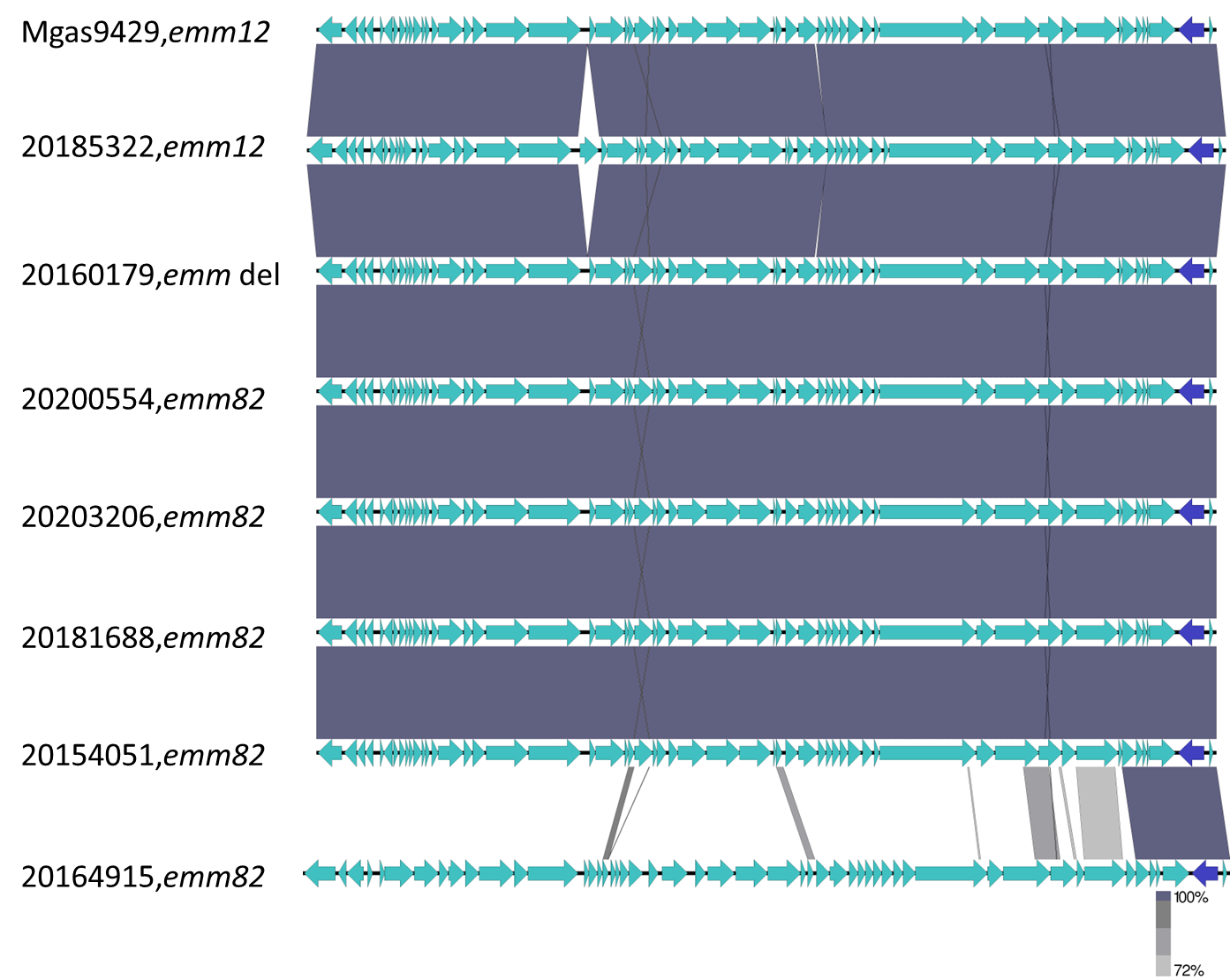
 **sFigure 4.** Prophages harboring *sda1* gene (blue arrow).

**
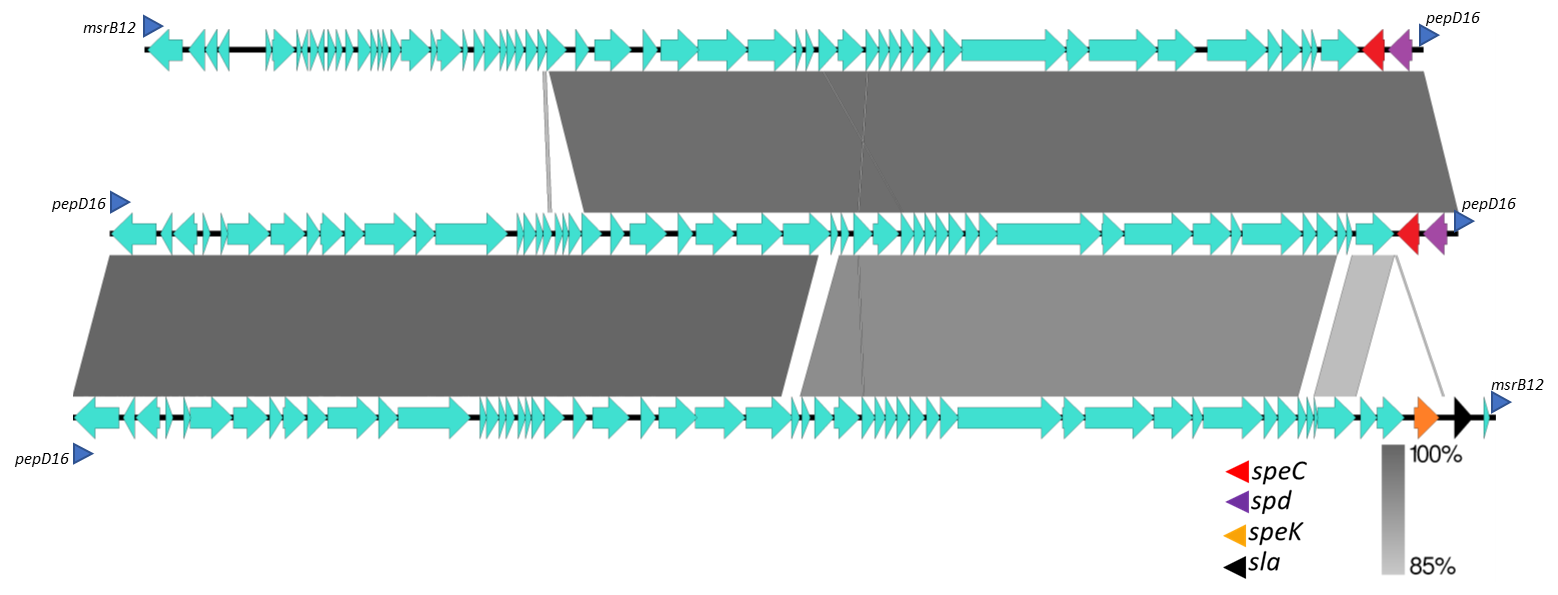
**

sFig. 5. Alignment of prophages carrying *speC/spd* and *speK/sla* from strain 20192362 with prophage carrying *speC/spd from strain 20200554.*


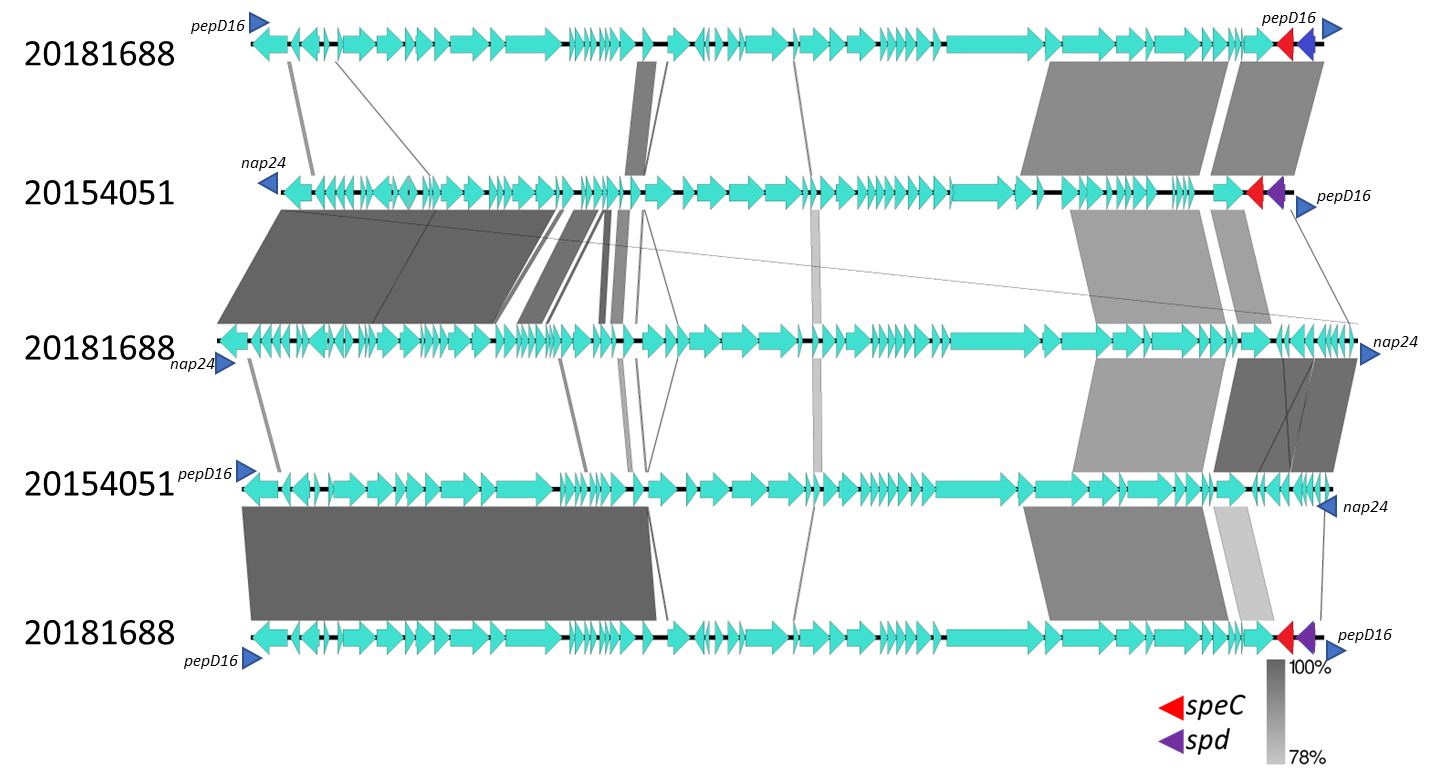


sFig. 6. Alignment of prophages from 54051 and 81688 that include *pepD16* and/or *nap24* flanking repeats


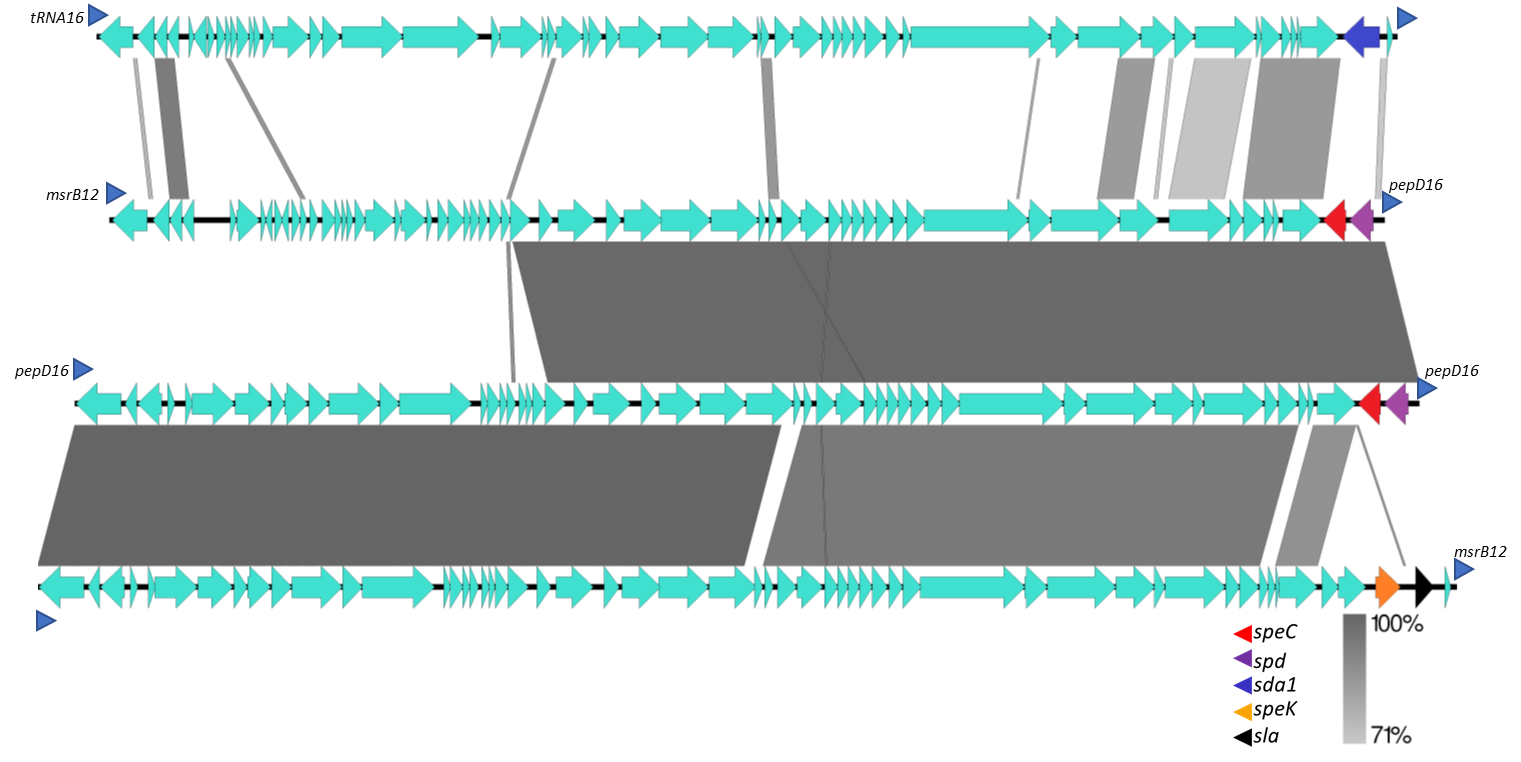


sFig. 7. Alignment of prophages carrying *speC/spd* and *speK/sla* from strain 20192362 with prophages carrying *sda1* and

*speC/spd* from strain 20200554.
